# Prevalent osteoporotic fractures in 622 obese and non- obese menopausal women

**Published:** 2015

**Authors:** C Poiana, M Carsote, V Radoi, A Mihai, C Capatina

**Affiliations:** * “C.I. Parhon” National Institute of Endocrinology, Bucharest, Romania; ** Department of Endocrinology, “Carol Davila” University of Medicine and Pharmacy, Bucharest, Romania; *** Obregia Hospital, Bucharest, Romania

**Keywords:** osteoporosis, fractures, obesity, body mass index, bone mineral density

## Abstract

**Hypothesis.** The osteoporotic fractures represent a worldwide economical issue. In order to prevent them, we need to understand the risk factors constellation. Although obesity was traditionally considered as protective against osteoporosis, recent data exposed an increased risk of falling and thus a high risk of some fractures.

**Objective.** We aimed to analyze the body mass index (BMI) in relationship with the bone mineral density (BMD) and the prevalent fractures.

**Methods and Results.** Between 2008 and 2014, a cross-sectional observational study included Romanian menopausal Caucasian women without a previous diagnosis of bone maladies, or specific anti-osteoporotic therapy. Prevalent fragility fractures were both self-declared and incidental vertebral. All the subjects had lumbar BMD (GE Lunar Prodigy DXA machine).

Out of 622 females (mean age of 58.65 years, mean BMI of 30.30 kg/ m2), 39.22% were obese (BMI ≥ 30kg/ m2). The fracture prevalence was 1.35% versus 1.67% in obese versus non-obese patients. The correlation coefficient between lumbar BMD and BMI was r=0.165, p<0.005. BMI in the fracture group was 31.68 kg/ m2 vs. 30.04 kg/ m2 in the non-fracture group (p=0.08). 15.91% of the entire cohort had prevalent fractures. Obesity prevalence among females with fractures was 30.3% versus 40.73% in the non-fracture group. The most frequent sites were distal forearm (42.42%) and vertebral (21.21%).

**Discussions & Conclusions.** Although the vertebral fractures might be underdiagnosed in our study and despite the fact that we enrolled a relatively young menopausal population, BMI positively correlated with BMD, regardless of the fractures’ prevalence. In early menopause, the most frequent fracture is distal forearm. BMI is higher in patients with prevalent fractures vs. non-fractures (borderline significance). Obesity might not protect from any type of fracture but future evidence is necessary since one third of osteoporotic fractures are met in women with a BMI ≥ 30kg/ m2.

## Background

Osteoporosis and fragility related fractures represent a worldwide problem [**1**]. The quality of life is closely related to the presence of osteoporosis and its complications as fractures [**2**]. Menopausal women represent the most important segment of population at risk. Yearly, new fracture risk factors are brought in front line based on scientific researches of both animal and cells lines models, including gene tests, moreover, relying on evidence based medicine regarding large human cohorts. Generally, it is recognized that body mass index (BMI) has a good positive correlation with bone mineral density (BMD) as assessed by central Dual-Energy X-Ray Absorptiometry (DXA) thus a very low BMI (like less than 18 kg/ m2) is an independent well known osteoporotic fracture risk [**3**]. On the other hand, the high values of BMI, although associating good values of BMD, are at high fracture risk based on an increased risk of fall, so obesity might not protect against all osteoporotic fractures as it was initially considered, although BMD is the major determinant of the fracture risk [**4**]. Another negative aspect of obesity is the recovery after fractures; it seems that a poorer outcome is found in obese versus non-obese population in orthopaedic trials [**5**]. Despite the traditional hypothesis that fat people do not suffer from osteoporosis mainly based on bone protective effect of relative hyperestrogenism, the last decades changed this optic and pointed out that obesity is in fact correlated to a higher risk of some fragility fractures. A higher weight increases the risk of falling, which cannot actually be prevented by anti-osteoporotic medication. Other common mechanisms that associate obesity to fractures involve inflammation and related cytokines, leptin pathways, adipokines, central and peripheral serotonin, etc. [**6**-**8**]. Other factors found in obese population targeting a higher risk of fracture are the following: an increased prevalence than seen in normal weighted population of type 2 diabetes mellitus, hypogonadism, insulin resistance, premature menopause, hypovitaminosis D, asthma, etc. [**9**]. The relationship between BMI and obesity on one hand, and fracture risk, prevalent fragility fractures and BMD on the other hand, is complex and not linear. A meta-analysis was published in 2014 regarding prospective studies with data originating from 25 countries (6457 hip fractures on 398,610 pre and postmenopausal women with an average age of 63 years who were followed up for 2.2 million person-years). 22% of these women were obese. More than 80% of osteoporotic (including hip) fractures were found in women with a BMI < 30 kg/ m2. Regardless of the adjusting for BMD, low BMI associates a risk of hip fracture and high BMI has a risk factor for elbow and humerus fractures [**10**]. Data provided by GLOW (Global Longitudinal study of Osteoporosis in Women) study provided self-reported analyses of fracture incidence during 3 years, on 52,939 women. 6.9% of the studied population reported a fracture: BMI is negatively correlated with vertebral, hip and wrist fractures; weight is positively linearly correlated with ankle fractures; for upper arm fractures only linear height was positively correlated; negative nonlinear weight and BMI correlation is found with rib and pelvic fractures at low BMI, and positive nonlinear correlation is showed at high BMI [**11**]. A specific risk for each fracture site would better describe the influence of BMI, especially in obese area. In this present study, we aimed to analyze the BMI in relationship to the BMD and prevalent fragility fractures in menopausal women.

## Material and Method

The study design was cross-sectional observational on Romanian menopausal Caucasian women. The patients were admitted for different medical reasons in the Department of Pituitary and Neuroendocrine Diseases from “C.I. Parhon” National Institute of Endocrinology, Bucharest, Romania (a tertiary centre of endocrinology), between 2008 and 2014. The patients were first evaluated based on anamnesis and if they fitted to the inclusion and exclusion criteria, they were further enrolled in the study. Some patients needed supplementary endocrine tests in order to check their eligibility and afterwards they were included in the study. The informed consent was obtained from each patient. The inclusion criteria were at least one year since the last menstruation (spontaneous or surgical menopause as cause of the secondary amenorrhea and no other endocrine or gynaecological cause). The exclusion criteria were previous diagnosis of bone diseases, including bone metastases, bone cancers, Paget’s disease, osteomalacia, rickets, osteogenesis imperfecta, previous diagnosis of osteoporosis (primary or secondary); previous or current medication targeting the fractures risk reduction as bisphosphonates, denosumab, strontium ranelate or bone anabolic medication as teriparatide; secondary causes of obesity as Cushing’s syndrome of any type (previous or current); hormonal replacement therapy at any moment in life; central DXA to provide unusable data. 

The clinical general and endocrine exam was performed by an endocrinologist. The Body Mass Index (BMI) was calculated based on the formula weight in kilograms (kg) divided to the square of height in meters squared (m2): BMI in kg/ m2. Normal weight was considered at a BMI between 18.5 and 24.9 kg/ m2. Obesity was considered at BMI ≥ 30 kg/ m2. The non-obese group was defined as any subject with a BMI < 30 kg/ m2. 

The personal medical history was considered based on the patients’ declarations and records from different hospitals (including circumstances of fall, trauma, fractures, and specific orthopaedic therapy for fractures). The prevalent fragility fractures were registered based on anamnesis (self-reported fractures), patients’ records (prevalent fractures). In selected cases (not in all), the incidental vertebral fractures were diagnosed based on profile lumbar X-Ray and very few patients had a thoracic – abdominal computed tomography performed for different medical reasons, and, in this manner, the incidental vertebral fractures were found. The lumbar profile X-Ray was not routinely performed only in cases with persistent back pain, loss of height since maximum achievement during lifespan and kyphosis. All the fractures considered in the study were fragility (osteoporotic, low trauma fractures) fractures and they were all found in menopause, being described as prevalent fractures (including newly diagnosed vertebral fractures). 

The GE Lunar Prodigy central DXA device was used to prove the Bone Mineral Density (BMD) at central sites lumbar, total hip and femoral neck. All the patients had a DXA examination at the same device. The BMD was expressed in grams divided to centimeters squared (g/ cm2). The WHO criteria for normal DXA, osteopenia, and osteoporosis were applied based on T-score [**12**]. SPSS 17 was used for a statistical analisis; the parameters analyzed were mean, standard deviation (SD), minimum (min), maximum (max), linear correlation coefficient (r) based on simple linear regression; statistically significance was considered at p value <0.05.

## Results

622 women were enrolled based on inclusion/ exclusion criteria. The anthropometric parameters were mean age of 58.65 years (max of 88 years); mean BMI of 30.3 kg/ m2; mean period of time since menopause of 12.29 years (ranges 1 to 59 years); mean lumbar BMD of 0.99 g/ cm2 (**Table 1**). 39.22% (N=244) of the entire cohort were obese. 1.35% of the obese patients versus 1.67% of the non-obese group had a prevalent fracture. 

**Table 1:** The parameters of the studied population (N=622)

parameter	mean	SD	min	max	units
age	58.65	8.64	41.00	88.00	years
BMI	30.30	6.55	18.40	50.00	kg/ m2
YSM	12.29	8.83	12.00	59.00	years
Lumbar BMD	0.99	0.19	0.49	1.52	g/ cm2
BMI=Body Mass Index; YSM=Years since Menopause; BMD=Bone Mineral Density (DXA)					

**Fig. 1:**
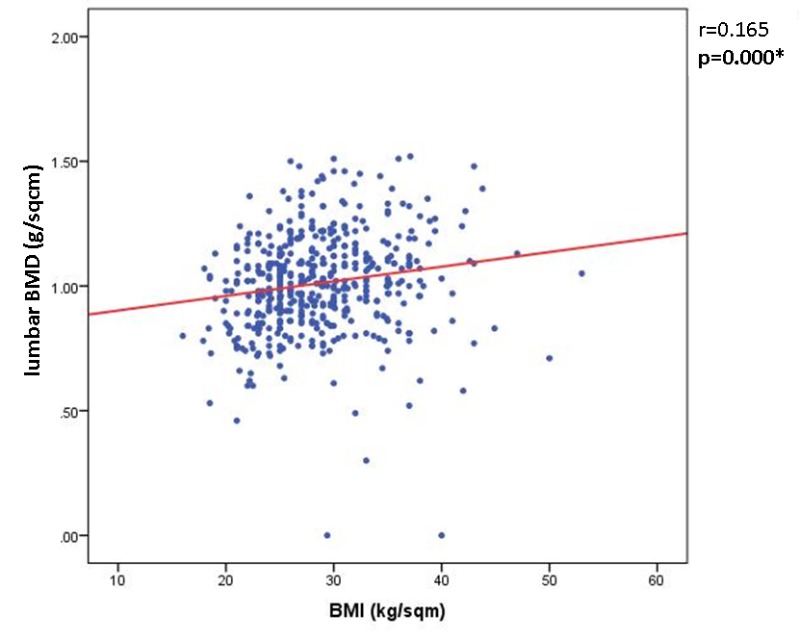
The Pearson correlation between BMI and lumbar BMD (r=0.165, p<0.0005)

The correlation coefficient between lumbar BMD and BMI for the all cohort was r=0.165, p<0.005 (Pearson Correlation); if the patients had no prevalent fragility fractures the value became r=0.15, p<0.005 (**Fig. 1**). The femoral neck BMD and BMI was r=0.154, p<0.005, regardless of the patients had or did not have prevalent fractures. The total hip BMD and BMI correlation is r=0.145, p<0.005 independent of fragility fractures prevalence.

99 (15.91%) out of 622 subjects had prevalent fractures. BMI was borderline statistically significant different between the group of females with fractures and without them (N=523, p=0.08) and age was also statistically significant: the fracture group had an average age of 61.68 years versus 58.1 years in fracture-free group, and also the lumbar BMD and the years since menopause (**Table 2**). 

**Table 2:** The parameters based on group with and without prevalent fractures

fractures	parameter	mean	SD	min	max	units
+ (N=99)	age	61.68	9.51	42.00	88.00	years
	BMI	31.68	7.25	18.40	50.41	kg/ m2
	YSM	15.80	10.23	1.00	59.00	years
	Lumbar BMD	0.92	0.17	0.60	1.46	g/ cm2
- (N=523)	age	58.10	8.35	36.00	84.00	years
	BMI	30.04	6.43	18.50	50.00	kg/ m2
	YSM	11.65	8.38	1.00	51.00	years
	Lumbar BMD	1.00	0.19	0.30	1.52	g/ cm2
BMI=Body Mass Index; YSM=Years since Menopause; BMD=Bone Mineral Density (DXA)					

**Fig. 2:**
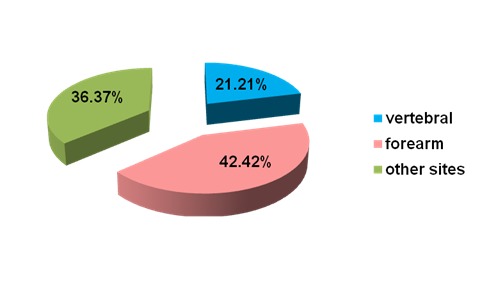
The fracture sites in prevalent fracture group

30.3% (N=30) of the fracture group were obese, while 40.73% (N=213) of the non-fracture group were obese. The most frequent fractures were distal forearm (N=42; 42.42% of all fractures); and vertebral (N=21, 21.21% of all prevalent fractures) (**Fig. 2**).

The other sites varied from tibia to arm, elbow, humerus, ribs, etc. at different sites; there was only one femoral neck fracture. 77.77% (N=77) of the prevalent fractures associated only a site of fracture (the spine was considered one site regardless of how many vertebras were involved). In the vertebral fracture group, the BMI was 31.5 kg/ m2 vs. 31.85 kg/ m2 in the non-vertebral fracture group (p=0.7).

40.9% of the vertebral fractures groups were obese; 29.58% were obese in the non-vertebral fracture group; 31.81% of the forearm fracture group were obese.

An analysis based on DXA scans and using the WHO criteria for defining osteoporosis found a group of subjects with lumbar BMD providing a T-score of ≤-2.5 (N=168 women) with a mean age of 60.83 years, a mean BMI of 31.2 kg/ m2, a mean menopausal period of time of 14.48 years (**Table 3**). 

**Table 3:** The parameters based on group with and without osteoporosis (based only on DXA T-Score)

Osteoporosis	parameter	mean	SD	min	max	units
+ (N=168)	age	60.83	8.54	41.00	78.00	years
	BMI	31.2	7.84	18.40	50.41	kg/ m2
	YSM	14.48	8.28	1.00	35.00	years
	Lumbar BMD	0.78	0.09	0.30	0.90	g/ cm2
- (N=454)	age	57.84	8.55	36.00	88.00	years
	BMI	29.78	6.05	18.50	50.00	kg/ m2
	YSM	11.47	8.89	1.00	59.00	years
	Lumbar BMD	1.07	0.16	0.46	1.52	g/ cm2
BMI=Body Mass Index; YSM=Years since Menopause; BMD=Bone Mineral Density (DXA)					

The females with obesity in T-score based osteoporosis group were 31.54% and 44.06% in the group of non-osteoporosis. The BMI was not different between the osteoporosis (N=168) and non-osteoporosis group (N=454, p value =0.44). The number of patients with prevalent fractures in osteoporosis group was N=38 (22.61%). The mean BMI of 34.35 kg/ m2 (within the sub-group with prevalent fractures and osteoporosis T-score) vs. 30.27 kg/ m2 (in the group with osteoporosis based on T-score but without prevalent fractures), was not statistically significant different (p=0.06). 

## Discussions

Based on our observations, the body mass index was positively correlated with the bone mineral density as provided by DXA. The results were independent of the prevalent fractures or the DXA site. This correlation coefficient was similar with data from literature although some authors reported different correlation coefficients depending on DXA site and a more precise association with weight better than BMI [**13**,**14**]. Almost 16% of the women had at least one prevalent fragility fracture. The percent was not so high probably because of the relatively young mean age of 58.65 years (early menopause) since the fracture incidence is age related [**15**]. Moreover, none of the subjects had a previous osteoporotic menopausal fracture; they remained without anti-osteoporosis therapy up to the moment when we collected the data for the study. Vitamin D and calcium supplements were allowed for the study and we did not use these for the statistical analysis because we did not have enough data to quantify the amount of supplements. The majority of the fractures were at the distal forearm (42.42%). The fact that spine fractures were less than expected for this age ranges, could be explained by the vertebral fractures probably underdiagnosed since no screening spine X-Ray or VFA (vertebral fracture assessment) was performed. 78.78% of the fractures were single (the spine was considered one site), which was an expected result for early menopause. The body mass index was not different between patients with or without fractures even if they had a T-score ≤ -2.5 at lumbar DXA. According to DXA, obesity was found in 30.3% of the females with prevalent fractures, and in 31.54% of the subjects with both fracture and osteoporosis. A similar cross-sectional study in Brazil, including 984 women aged ≥ 55 years with an average age of 67.1 years and an average body mass index of 29.2 kg/ m2 found a similar percent of fractures in obese and non-obese subjects (17.3% vs. 16.0%), and that 41.4% of fragility fractures occurred in obese females [**16**].

Our study had some limits. There was no consistent screening of the vertebral fractures, so a number of asymptomatic vertebral fractures was undetected; the majority of the fractures were self reported. The data were collected from a single tertiary centre, so there were some limits when extending the data to the entire population. There was no quantification of the time since fracture in cases of non-incidental fractures and we had no data on the fracture healing time. Potential confounders factors that might influence the risk of fracture (including the risk of fall) especially in obese population, as vitamin D deficit, type 2 diabetes mellitus, depression and the use of specific medication for depression, were not taken into account. We did not have the exact weight of the patient at the moment of a fall related fracture except for the newly diagnosed spine fractures (incidental fractures) that we detected on admission. 

## Conclusions

Based on our cross-sectional observations in a menopausal group of 622 subjects, without previous targeted therapy for osteoporosis, we concluded that the body mass index is positively statistically significant correlated with the lumbar bone mineral density, regardless of the prevalent fragility fractures, and is higher in patients with prevalent fractures vs. without fractures, including the sub-analyses for osteoporotic subjects according to T-score (borderline significance). The most frequent fractures are forearm (at a mean age group of 58.65 years). Obesity might not protect from any type of fracture and future evidence is necessary since the obese women carry an important fracture burden underling one third of the prevalent osteoporotic fractures.

**Conflict of interest:**

None

**Acknowledgement:** We would like to thank the women included in this study and also all the medical team involved in collecting the data and performing the DXA scans and the X-Ray evaluations. 
